# Serum free triiodothyronine is inversely associated with diabetic peripheral neuropathy but not with carotid atherosclerotic lesions in euthyroid patients with type 2 diabetes

**DOI:** 10.1186/s13098-021-00760-2

**Published:** 2021-12-04

**Authors:** Mei-Fang Li, Jiang-Feng Ke, Shuai Li, Jun-Wei Wang, Zhi-Hui Zhu, Jing-Bo Li

**Affiliations:** 1 Department of Emergency, Shanghai Jiao Tong University Affiliated Sixth People’s Hospital, Shanghai, China; 2 Department of Endocrinology and Metabolism, Shanghai Jiao Tong University Affiliated Sixth People’s Hospital, Shanghai Clinical Medical Center of Diabetes, Shanghai Key Clinical Center of Metabolic Diseases, Shanghai Institute for Diabetes, Shanghai Key Laboratory of Diabetes, Shanghai, China; 3 Department of Cardiology, Shanghai Jiao Tong University Affiliated Sixth People’s Hospital, 600 Yishan Road, Shanghai, 200233 China

**Keywords:** Free triiodothyronine, Diabetic peripheral neuropathy, Carotid atherosclerotic lesions, Type 2 diabetes, Euthyroid function

## Abstract

**Background:**

The associations between serum free triiodothyronine (FT3) and diabetic peripheral neuropatprohy (DPN)/carotid atherosclerotic lesions in euthyroid patients with type 2 diabetes are still unclear. The purpose of our study was to explore the relations of FT3 to DPN and carotid atherosclerotic lesions in Chinese type 2 diabetes inpatients with euthyroid function.

**Methods:**

2477 euthyroid inpatients with type 2 diabetes were recruited and they were stratified into quartiles by FT3 levels in this cross-sectional study. Peripheral neuropathy was assessed by neurological symptoms and signs as well as nerve conduction velocity tests. Carotid atherosclerotic lesions, including carotid intima-media thickness, plaque and stenosis, were evaluated by Doppler ultrasound.

**Results:**

The prevalence of DPN in type 2 diabetic patients exhibited the significant decrease across the FT3 quartiles (23.5%, 20.9%, 18.8%, and 11.2%, respectively, p < 0.001). Multiple logistical regression analysis also revealed that FT3 quartiles were significantly and inversely associated with DPN. Compared with the subjects in the highest FT3 quartile, the adjusted odds ratios (95% confidence interval) of DPN from the first to third FT3 quartile were successively 2.338 (1.407–3.884), 1.903 (1.134–3.194) and 1.598 (0.960–1.125). The patients with DPN had significantly higher prevalence of carotid atherosclerotic lesions compared with non-DPN patients. However, no statistical association was observed between FT3 quartiles and carotid atherosclerotic lesions after adjusting for confounder factors.

**Conclusions:**

Lower FT3 within the normal range was independently associated with DPN, but not with carotid atherosclerotic lesions in Chinese euthyroid inpatients with type 2 diabetes.

**Supplementary Information:**

The online version contains supplementary material available at 10.1186/s13098-021-00760-2.

## Background

Thyroid dysfunction, whether overt or subclinical, has been widely reported to closely associate with the heart and cardiovascular deteriorations such as coronary artery disease (CAD), atrial fibrillation and heart failure [1–4]. It has also been reported that subclinical hypothyroidism (SCH) is strongly related with diabetic vascular complications, including CAD, diabetic nephropathy (DN) and retinopathy (DR) [5–7]. For example, Chen et al. [7] demonstrated that SCH in Type 2 diabetics suffered from roughly 3.15-fold risk of DN in the cross-sectional analysis and had 2.93-fold risk for cardiovascular events during the 44.0-months follow-up compared with euthyroid diabetics. However, the studies on the thyroid dysfunction and diabetic peripheral neuropathy (DPN) were extremely scarce.

Recently, it has also been discovered that alterations in thyroid function, even within reference ranges, relate to atherosclerosis for general population and angina patients [8–10]. The Cardiovascular Health Study and the Rotterdam Study found that euthyroid participants faced the higher risk of heart failure and sudden cardiac death along with the growing free thyroxine (FT4) levels [10, 11]. However, free triiodothyronine (FT3) as an active metabolite of thyroid hormones was not evaluated in these studies, and the data regarding the relation between FT3 and atherosclerosis were limited.

Furthermore, several recent studies have reported that close relations are observed between low-normal FT3 levels and type 2 diabetic macrovascular and microvascular complications [12, 13]. For example, Wu et al. [13] revealed that the occurrence of DN exhibited gradual reduction across the FT3 tertile groups (59.6%, 46.4%, and 38.6%, p < 0.01) in type 2 diabetes with euthyroid function, and FT3 levels were negatively correlated with urinary albumin-to-creatinine ratio after adjustment for various risk factors according to multiple linear regression analysis.

However, so far there is no data on the association of FT3 with DPN and carotid atherosclerotic lesions in type 2 diabetic patients with normal thyroid function. Therefore, in our study we tried to clarify whether there existed some relations between serum FT3 levels within the normal range and the presence of DPN and carotid atherosclerotic lesions in Chinese type 2 diabetic patients with euthyroid function.

## Materials and methods

### Subjects and study design

**Fig. 1:**
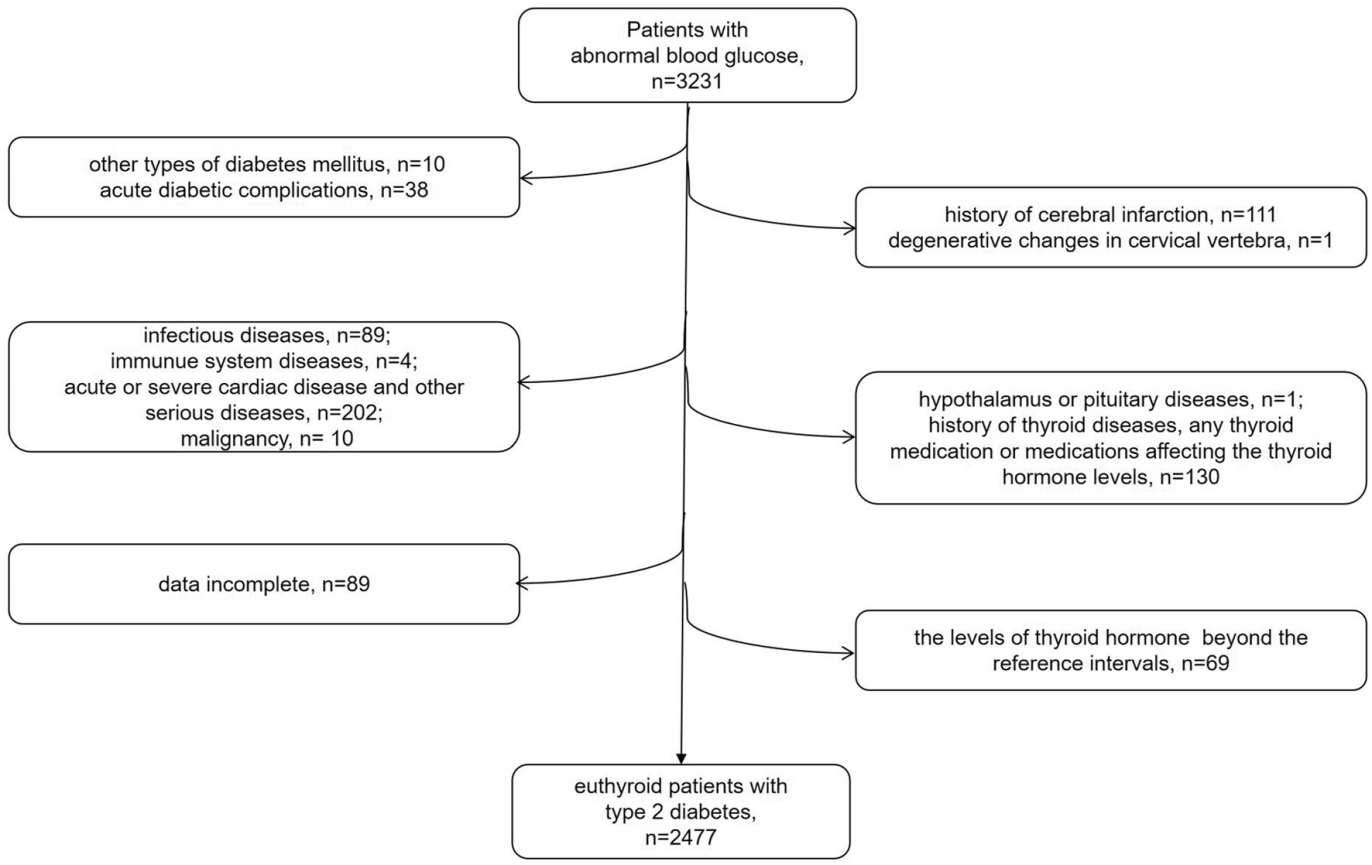
A flow chart of patient enrollment

3231 patients with abnormal blood glucose who hospitalized in the Endocrinology and Metabolism Department of Shanghai Jiao Tong University Affiliated Sixth People’s Hospital from June 2005 to May 2012 were collected. The diagnosis of type 2 diabetes was performed according to the World Health Organization standards in 1999 and American Diabetes Association criteria in 2012 [14]. And 754 patients were excluded as the following reasons: (1) other types of diabetes mellitus or acute diabetic complications; (2) history of cerebral infarction, degenerative changes in cervical vertebra; (3) infectious diseases, immunue system diseases, acute or severe cardiac diseases and other serious diseases, malignancy and pregnancy; (4) hypothalamus or pituitary diseases, history of thyroid diseases, any thyroid medication (levothyroxine or antithyroid drugs), or medications that affected the thyroid hormone levels; (5) lack of thyroid function, neuropathy assessment, carotid ultrasound examination, and complete clinical data; (6) the levels of thyroid stimulating hormone (TSH), FT3 and FT4 beyond the reference intervals (0.27–4.2 mIU/L for TSH, 3.10–6.80 pmol/L for FT3, and 12–22 pmol/L for FT4 in this study). Ultimately, 2477 type 2 diabetic patients with euthyroid function were included in our present analysis. Written informed consents were got from the whole participants. The flow chart of the subject enrollment in our study showed in Fig. 1. Our cross-sectional study was approved by the Human Research and Ethics Committee of Shanghai Jiao Tong University Affiliated Sixth People’s Hospital and adhered to the tenets of the Declaration of Helsinki.

### Physical examination and laboratory measurements

Height, weight, waist circumference, hip circumference and blood pressure of all participants were measured. The body mass index (BMI) was obtained through the weight (kg) divided by the square of height (m). Waist hip ratio (WHR) was calculated as the waist circumference (cm) divided by the hip circumference (cm). Venous blood samples were drawn for the determination of glycosylated hemoglobin A1c (HbA1c), fasting plasma glucose (FPG), 2 h postprandial plasma glucose (2 h PPG), fasting C-peptide (FCP), 2 h postprandial C-peptide (2 h PCP), total triglycerides (TTG), total cholesterol (TC), high-density lipoprotein cholesterol (HDL-C), low-density lipoprotein cholesterol (LDL-C), alanine aminotransferase (ALT), Serum creatinine (Scr), serum uric acid (SUA), C-reactive protein (CRP) and thyroid function profile after an overnight fast. Thyroid function profile, including FT3, FT4 and TSH, were measured using a chemiluminescence technique (Cobas 6000; Roche Diagnostics GmbH, Mannheim, Germany). Urine albumin-to-creatinine ratio (UACR) was collected from three isolated morning urine samples during the hospitalization and was expressed as the average of three samples in our present study. The homeostasis model assessment for insulin resistance (HOMA-IR) was determined using the HOMA calculator version 2.2.3 [15]. The estimated glomerular filtration rate (eGFR) was calculated using simplified MDRD formula: estimated GFR (eGFR) = 186.3 × (Serum creatinine)^−1.154^ × (age)^−0.203^(× 0.742 if female) [16].

### Neuropathy assessment

Assessment of neuropathy, including neurological symptoms and signs as well as nerve conduction velocity tests, were conducted to the whole subjects. Firstly, following the Toronto Clinical Scoring System [17], the licensed physicians evaluated the patients’ neurological symptoms and signs, and recorded as positive when the patients had any feelings including pain, numbness, tingling, weakness of foot, reflex abnormalities or ataxia. Secondly, nerve conduction velocity tests were conducted by electromyogram. Briefly, electromyogram was applied to measure the nerve conduction velocities (NCVs) of patients’ bilateral median, ulnar, tibia, common peroneal and superficial peroneal nerves while they remained calm and relaxed with their local skin temperature kept constant (> 31 ℃) by an infrared lamp. And considering Chinese people’s NCV reference value, we defined the threshold for lowered NCVs as used [18].

### Carotid ultrasonography measurements

Carotid ultrasonography was performed through a machine with a phased-array transducer (Acuson Sequoia 512, Siemens) and was conducted by the certified and skilled sonographers. The ultrasound scanning protocol adopted in this study was further modified from procedures used in previous studies [19–21]. That is, the sonographers successively recorded and read the bilateral images of the common carotid arteries (1 cm proximal to the dilatation of the carotid bulb), the carotid bulb (identified by the absence of the parallel wall in the common carotid artery), and the internal carotid artery (1 cm distal to the tip of the flow divider that separates the external and internal carotid arteries) to detect carotid plaques and stenosis. The intima-media thickness (IMT) was the distance between the lumen-intima interface and the media-adventitia interface [19].

### Diagnostic criteria

DPN was diagnosed as the patients had both obviously clinical DPN features (defined as at least two positive findings among sensory symptoms, signs or abnormal reflections in accordance with symmetrical polyneuropathy at distal end) and abnormal results on nerve conduction tests [defined by the presence of at least one abnormal nerve attribute (of amplitude, latency, F-wave, or NCV) in two or more nerves among the median, peroneal, and sural nerves] described in detail previously [22].

Carotid IMT (CIMT) was defined as the mean of the left and right IMTs of the common carotid artery. According to the Mannheim consensus [23], carotid plaques were defined as focal structures encroaching into the arterial lumen of 0.5 mm or 50% of the surrounding IMT value or IMT of > 1.5 mm. Carotid stenosis was defined as any degree of narrowing in the carotid arteries caused by carotid plaques [20, 21].

### Statistical analyses

For statistical analysis, SPSS v. 16.0 was used. Figures were created by GraphPad Prism 5.0. Data were expressed as either mean ± standard deviation or medians (interquartile range 25–75%) for continuous variables and percentages (%) for categorical variables. For continuous variables, normality was tested by P–P plot. One-way ANOVAs with LSDs were used for normal distributions and Kruskal–Wallis H tests were used for skewed distributions. The Chi-squared test was used to compare categorical values. Stepwise multiple binary logistic regression and linear regression analyses were performed to evaluate the relations of the FT3 quartiles to the odds ratios (ORs) of DPN, carotid plaque and stenosis. All p values were two-tailed, and p < 0.05 was considered as statistically significant.

## Results

### Characteristics of the subjects according to FT3 quartiles

Of the 2477 participants analyzed, the mean age of them was 59 ± 11 years and 1390 patients (56.1%) were men. The prevalence of DPN, carotid plaques and carotid stenosis was 18.6%, 47% and 0.6%, and the mean CIMT value was 0.84 ± 0.18 mm.

The clinical characteristics of all the patients grouped by FT3 quartiles are presented in Table 1. The patients were stratified into quartiles by the FT3 levels with the cutoff limits of 3.10–4.10, 4.10–4.40, 4.40–4.72, and 4.72–6.80 pmol/L. The patients in the higher FT3 quartiles were younger and more likely to be male. And the patients in the higher FT3 quartiles had higher levels of BMI, DBP, FCP, 2 h PCP, TTG, Scr, FT4, and lower 2 h PPG, HbA1c, TC, UACR, and CRP (all p < 0.05) even after adjustment for age and sex (Additional file 1: Table S1).

**Table 1:** Clinical characteristics of the subjects

Variables	Q1 (n = 612)	Q2 (n = 616)	Q3 (n = 622)	Q4 (n = 627)	p value
FT3 (pmol/L)	3.10–4.10	4.10–4.40	4.40–4.72	4.72–6.80	–
Age (years)	64 ± 11	61 ± 11	58 ± 12	55 ± 11	< 0.001
Men (n, %)	261 (42.6%)	298 (48.4%)	372 (59.8%)	459 (73.2%)	< 0.001
DD (months)^a^	120 (48–180)	96 (48–156)	90 (36–144)	72 (24–120)	< 0.001
Smoking (n, %)	114 (18.6%)	133 (21.6%)	160 (25.7%)	241 (38.4%)	< 0.001
Alcohol (n, %)	48 (7.8%)	71 (11.5%)	85 (13.7%)	114 (18.2%)	0.002
Hypertension (n, %)	314 (51.3%)	330 (53.6%)	300 (48.2%)	270 (43.1%)	0.001
BMI (kg/m^2^)	24.63 ± 3.68	25.28 ± 3.38	25.08 ± 3.45	25.41 ± 3.38	0.001
WHR	0.93 ± 0.35	0.92 ± 0.07	0.91 ± 0.06	0.92 ± 0.06	0.378
SBP (mmHg)	133 ± 18	132 ± 17	132 ± 18	131 ± 16	0.016
DBP (mmHg)	79 ± 10	79 ± 10	80 ± 9	82 ± 9	< 0.001
FPG (mmol/l)^a^	7.55 (6.04–9.82)	7.82 (6.31–10.08)	7.90 (6.26–9.69)	7.81 (6.40–9.63)	0.481
2 h PPG (mmol/l)	14.13 ± 4.96	14.07 ± 4.66	13.89 ± 4.66	13.67 ± 4.72	0.070
HbA1c (%)^a^	9.3 (7.5–11.5)	8.7 (7.3–10.5)	8.5 (7.3–10.2)	8.4 (7–9.9)	< 0.001
FCP (ng/mL)^a^	1.57 (0.89–2.40)	1.73 (1.10–2.54)	1.82 (1.20–2.53)	1.89 (1.30–2.53)	0.020
2 h PCP (ng/mL)	3.75 ± 2.87	4.15 ± 2.74	4.46 ± 3.01	4.75 ± 2.71	< 0.001
HOMA-IR^a^	4.94 (2.98–8.40)	4.88 (2.96–7.85)	4.59 (2.91–7.75)	4.40 (2.72–7.40)	0.292
TTG (mmol/L)^a^	1.38 (0.92–2.08)	1.50 (1.01–2.18)	1.54 (1.04–2.41)	1.51 (1.09–2.10)	0.838
TC (mmol/L)	4.88 ± 1.35	4.84 ± 1.33	4.79 ± 1.04	4.67 ± 0.99	0.001
HDL-C (mmol/L)	1.17 ± 0.40	1.14 ± 0.31	1.10 ± 0.31	1.10 ± 0.28	< 0.001
LDL-C (mmol/L)	3.16 ± 1.03	3.18 ± 0.98	3.24 ± 0.93	3.17 ± 0.92	0.512
ALT (U/l)^a^	17 (12–26)	19 (13–29)	20 (14–30)	20 (14–30)	0.017
Scr (μmol/l)^a^	63 (52–80)	65 (55–77)	65 (55–78)	67 (56–78)	0.014
SUA (μmol/l)^a^	310 (251–369)	300 (248–364)	310 (265–377)	319 (267–377)	0.023
UACR (mg/g)^a^	13.05 (6.56–40.28)	11.73 (6.77–32.96)	11.25 (6.51–27.99)	10.14 (5.99–24.68)	0.024
eGFR (ml/min/1.73 m^2^)^a^	95.35 (76.97–118.23)	101.37 (83.68–117.81)	102.78 (86.18–120.92)	108.25 (92.07–126.77)	< 0.001
CRP (mg/L)^a^	1.46 (0.53–3.87)	1.09 (0.54–2.61)	0.96 (0.47–2.12)	0.93 (0.45–1.87)	< 0.001
FT4 (pmol/L)	15.96 ± 2.19	16.01 ± 2.20	16.34 ± 2.19	16.74 ± 2.21	< 0.001
TSH (mIU/L)	1.74 ± 0.89	1.74 ± 0.81	1.69 ± 0.84	1.67 ± 0.85	0.405

Values are expressed as the mean ± standard deviation, median with interquartile range, or percentages

*FT3* free triiodothyronine, *DD* duration of diabetes, *BMI* body mass index, *WHR* waist hip ratio, *SBP* systolic blood pressure, *DBP* diastolic blood pressure, *FPG* fasting plasma glucose, *2 h PPG* 2 h postprandial plasma glucose, *HbA1c* glycosylated haemoglobin A1c, *FCP* fasting C-peptide, *2 h PCP* 2-h postprandial C-peptide, *HOMA-IR* homeostatic model assessment of insulin resistance, *TTG* total triglyceride, *TC* total cholesterol, *HDL-C* high-density lipoprotein cholesterol, *LDL-C* low-density lipoprotein cholesterol, *ALT* alanine aminotransferase, *Scr* serum creatinine, *SUA* serum uric acid, *UACR* urine albumin-to-creatinine ratio, *eGFR* estimated glomerular filtration rate, *CRP* C-reactive protein, *FT4* free thyroxine, *TSH* thyroid stimulating hormone

^a^Non-normal distribution of continuous variables

### Comparison of DPN among the FT3 quartile groups

**Fig. 2:**
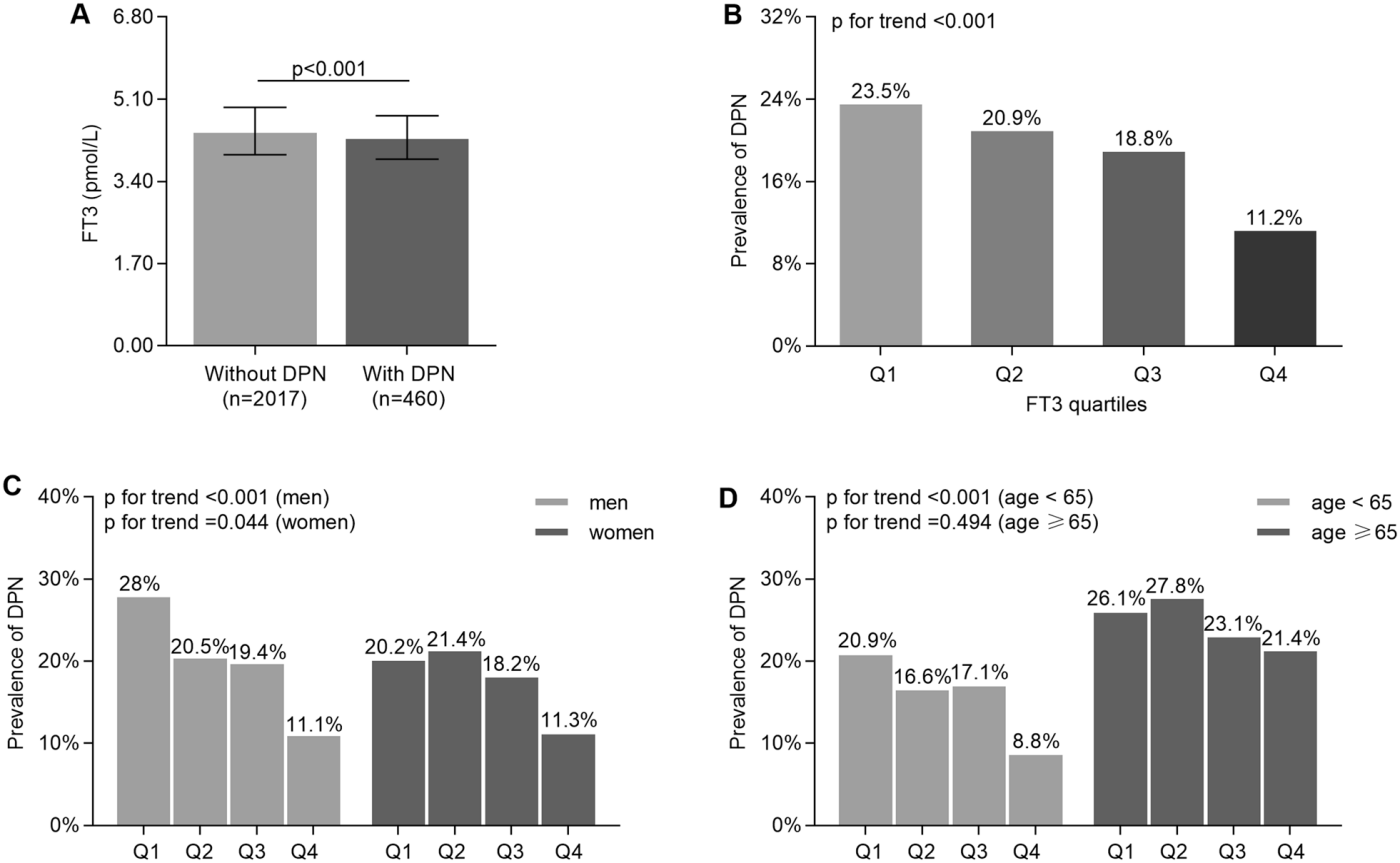
Comparison of DPN among the FT3 quartile groups. **A** Comparison of the FT3 levels between the diabetics with and without DPN. **B** Comparison of the prevalence of DPN among the four groups. **C** Comparison of the prevalence of DPN in FT3 quartile groups divided by sex. **D** Comparison of the prevalence of DPN in FT3 quartile groups stratified by age. Q1 (quartile1, FT3 3.10–4.10 pmol/L), Q2 (quartile2, FT3 4.10–4.40 pmol/L), Q3 (quartile3, FT3 4.40–4.72 pmol/L), Q4 (quartile4, FT3 4.72–6.80 pmol/L)

Figure 2 illustrates the comparison of DPN among the FT3 quartiles in type 2 diabetic patients. Patients with DPN yielded significantly lower FT3 levels compared with those without DPN (p = 0.001; Fig. 2A). The prevalence of DPN significantly decreased with the increasing FT3 quartile in type 2 diabetic patients (23.5%, 20.9%, 18.8%, and 11.2%, respectively, p for trend < 0.001; Fig. 2B). In the gender stratification, the prevalence of DPN performed significant decreasing trend with the increase of the FT3 quartiles both in men and women (p for trend < 0.001 and = 0.044, respectively, Fig. 2C). Interestingly, in the age stratification, the remarkable decrease in the prevalence of DPN was only found in patients with age < 65 (p for trend < 0.001, Fig. 2D).

### Comparison of carotid atherosclerotic lesions among the FT3 quartiles

**Fig. 3:**
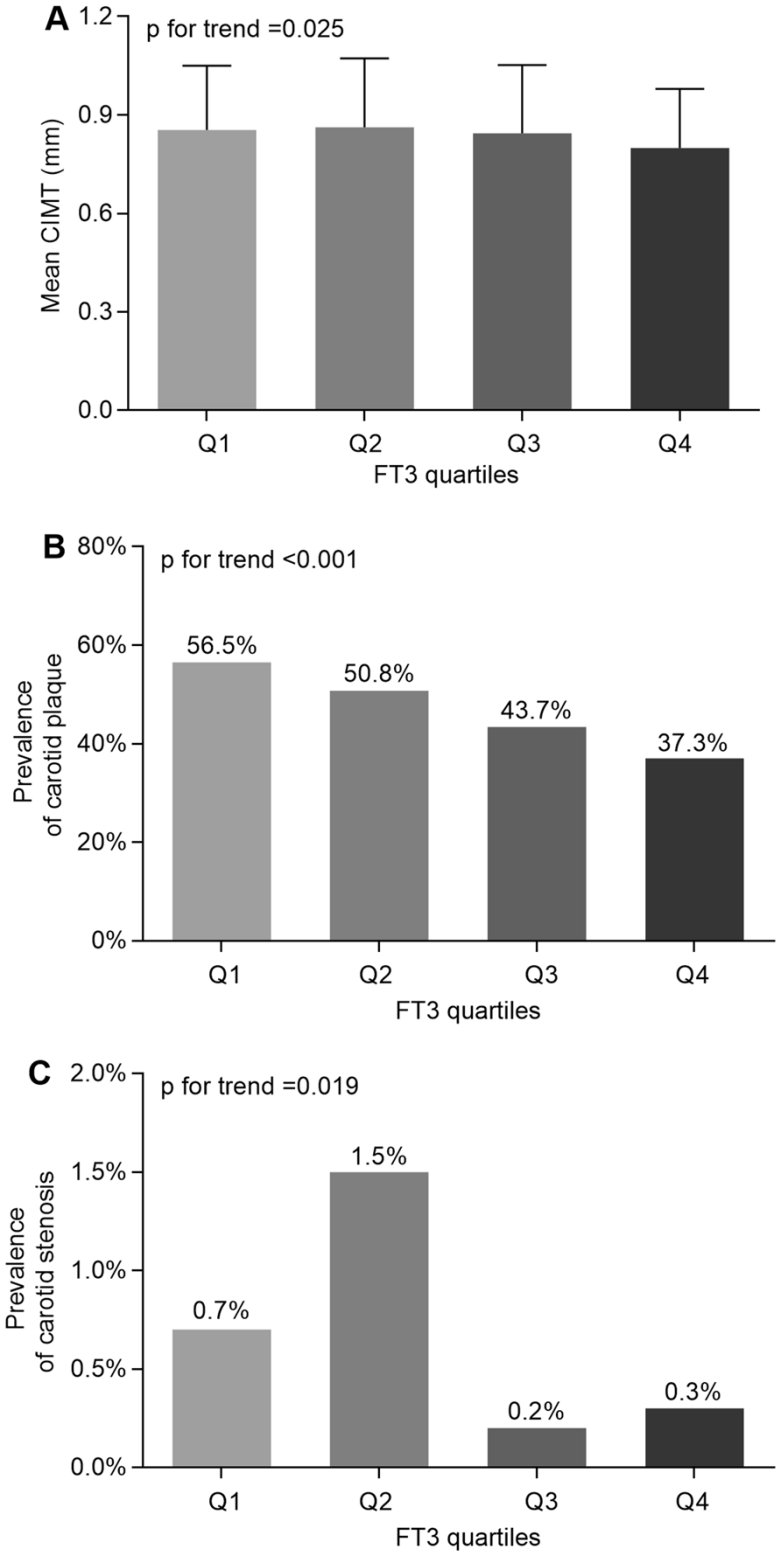
Comparison of carotid atherosclerotic lesions among FT3 quartile groups. **A** Comparison of mean CIMT among the four groups. **B** Comparisons of the prevalence of carotid plaque among four groups. **C** Comparison of the prevalence of carotid stenosis among the four groups

A comparison of the atherosclerotic lesions among the FT3 quartile groups is shown in Fig. 3. Significantly lower values of CIMT (0.85 ± 0.19 mm, 0.86 ± 0.21 mm, 0.84 ± 0.21 mm, and 0.79 ± 0.18 mm, respectively) (Fig. 3A) and a lower prevalence of carotid plaque (Fig. 3B) were found across the increasing FT3 quartiles. Furthermore, the prevalence of carotid stenosis also exhibited a dramatical difference among the four groups (Fig. 3C).

### Associations of FT3 quartiles with DPN and carotid atherosclerotic lesions

Table 2 presents the associations between the FT3 quartiles and the presence of DPN in type 2 diabetes. In the Model 1, the FT3 quartiles were independently associated with a decreased prevalence of DPN (p for trend = 0.001). After adding other clinical indicators (Model 2 and 3), the FT3 quartiles remained an independent correlation with a decreased prevalence of DPN (p for the trends = 0.001 and 0.01 in Model 2 and 3, respectively). Accordingly, the patients from the lowest to third FT3 quartile had 2.338-, 1.903-, and 1.598-fold risk of DPN, respectively, relative to those in the highest quartile.

**Table 2:** Association of the FT3 quartiles with DPN in type 2 diabetics

	ORs (95% CI)				p value for trend
	Q4	Q3	Q2	Q1	
Model 1	1 (ref.)	1.648 (1.101–2.468)	1.867 (1.238–2.814)	2.315 (1.530–3.503)	0.001
Model 2	1 (ref.)	1.746 (1.152–2.646)	1.985 (1.300–3.031)	2.445 (1.589–3.763)	0.001
Model 3	1 (ref.)	1.598 (0.960–1.125)	1.903 (1.134–3.194)	2.338 (1.407–3.884)	0.010

Model 1: age, sex, smoking, alcohol and hypertension

Model 2: Model 1 + DD, BMI, WHR, SBP and DBP

Model 3: Model 2 + HbA1c, ALT, eGFR, SUA, UACR, TTC, TG, HDL-C, LDL-C, FPG, 2 h PPG, FCP, 2 h PCP, HOMA-IR, CRP, FT4 and TSH

*Q1* (quartile 1, FT3 3.10–4.10 pmol/L), *Q2* (quartile 2, FT3 4.10–4.40 pmol/L), *Q3* (quartile 3, FT3 4.40–4.72 pmol/L), *Q4* (quartile 4, FT3 4.72–6.80 pmol/L), *OR* odds ratio, *CI* confidence interval, *DD* duration of diabetes, *BMI* body mass index, *WHR* waist hip ratio, *SBP* systolic blood pressure, *DBP* diastolic blood pressure, *HbA1c* glycosylated haemoglobin A1c, *ALT* alanine aminotransferase, *eGFR* estimated glomerular filtration rate, *SUA* serum uric acid, *UACR* urine albumin-to-creatinine ratio, *TTG* total triglyceride, *TC* total cholesterol, *HDL-C* high-density lipoprotein cholesterol, *LDL-C* low-density lipoprotein cholesterol, *FPG* fasting plasma glucose, *2 h PPG* 2 h postprandial plasma glucose, *FCP* fasting C-peptide, *2 h PCP* 2-h postprandial C-peptide, *HOMA-IR* homeostatic model assessment of insulin resistance, *CRP* C-reactive protein, *FT4* free thyroxine, *TSH* thyroid stimulating hormone

Table 3 demonstrates the associations of FT3 quartiles with carotid atherosclerotic lesions. Surprisingly, the remarkable associations of FT3 quartiles and carotid atherosclerotic lesions disappeared when stepwise multiple binary logistic regression and linear regression analyses were carried out (Model 1 and 2). After adding other potential confounders (Model 3), no significant correlations were found between FT3 quartiles and carotid atherosclerotic lesions.

**Table 3:** Association of the FT3 quartiles with carotid atherosclerotic lesions in type 2 diabetics

	Mean CIMT		Carotid plaques		Carotid stenosis	
	β	p value	OR	P value	OR	p value
Model 1	–	0.774	–	0.487	–	0.211
Model 2	–	0.746	–	0.615	–	0.211
Model 3	–	0.624	–	0.845	–	0.546

Model 1: age, sex, smoking, alcohol and hypertension

Model 2: Model 1 + DD, BMI, WHR, SBP and DBP

Model 3: Model 2 + HbA1c, ALT, eGFR, SUA, UACR, TTC, TG, HDL-C, LDL-C, FPG, 2 h PPG, FCP, 2 h PCP, HOMA-IR, CRP, FT4 and TSH

*OR* odds ratio, *DD* duration of diabetes, *BMI* body mass index, *WHR* waist hip ratio, *SBP* systolic blood pressure, *DBP* diastolic blood pressure, *HbA1c* glycosylated haemoglobin A1c, *ALT* alanine aminotransferase, *eGFR* estimated glomerular filtration rate, *SUA* serum uric acid, *UACR* urine albumin-to-creatinine ratio, *TTG* total triglyceride, *TC* total cholesterol, *HDL-C* high-density lipoprotein cholesterol, *LDL-C* low-density lipoprotein cholesterol, *FPG* fasting plasma glucose, *2 h PPG* 2 h postprandial plasma glucose, *FCP* fasting C-peptide, *2 h PCP* 2-h postprandial C-peptide, *HOMA-IR* homeostatic model assessment of insulin resistance, *CRP* C-reactive protein, *FT4* free thyroxine, *TSH* thyroid stimulating hormone

### Comparisons of carotid atherosclerotic lesions between the diabetics with and without DPN

**Fig. 4:**
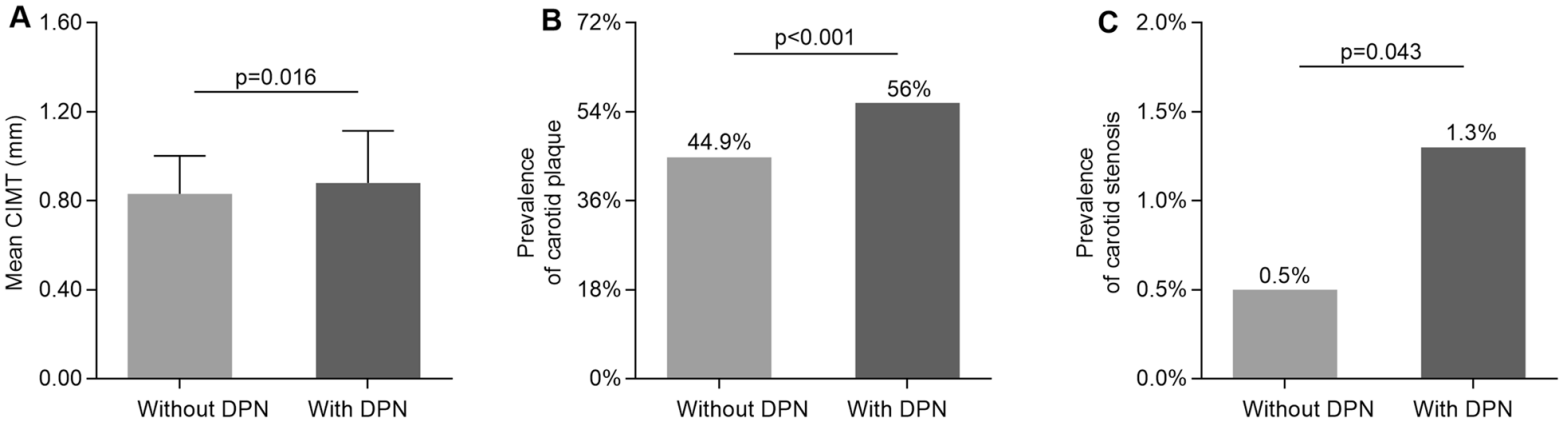
Comparison of carotid atherosclerotic lesions between the diabetics with and without DPN. **A** Comparison of the mean CIMT value between the diabetics with and without DPN. **B** Comparison of the prevalence of carotid plaque between the diabetics with and without DPN. **C** Comparison of the prevalence of carotid stenosis between the diabetics with and without DPN

The comparisons of carotid atherosclerotic lesions in type 2 diabetic patients with and without DPN are illustrated in Fig. 4. Patients with DPN had a higher value of CIMT (0.88 ± 0.23 mm vs. 0.83 ± 0.17 mm), and a higher prevalence of carotid plaque and stenosis when comparing with non-DPN patients (Fig. 4A–C).

## Discussion

Recent studies have suggested alternations in thyroid hormone levels within the normal range are valuable predictors for adverse cardiac events; So far, little literature reported the association between FT3 and carotid atherosclerosis. Furthermore, to our knowledge, no studies have investigated the relations of FT3 and DPN in individuals with normal thyroid function as of now.

In order to fill this gap in the literature, we undertook an analysis to explore whether there existed relations among FT3, DPN and carotid atherosclerotic lesions in Chinese type 2 diabetic patients with normal thyroid function. We found that the patients with DPN yielded lower FT3 levels compared with those without DPN. Moreover, the prevalence of DPN gradually decreased across FT3 quartiles. And the logistic regression analysis also revealed that the FT3 quartiles were inversely correlated with DPN even after adjustment for various risk factors. However, no significant difference was observed between FT3 and carotid atherosclerotic lesions after adjusting for confounders. Therefore, our results suggested that serum FT3 levels exhibited a potentially significant association with DPN in euthyroid patients with type 2 diabetes.

DPN, as the most common complication of diabetes, affects about half of patients and inevitably leads to reduced quality of life and increased socioeconomic burden [24, 25]. Therefore, it is quite necessary to prevent and treat DPN. Nowadays, researchers have made great progress in the study of the DPN pathophysiological mechanisms, but its underlying mechanisms have not yet been completely elucidated. Furthermore, with the role of thyroid hormones in organism physiology and functional development well studied, researchers start to work on the relations of thyroid hormones to diabetic microvascular complications. In a recent study by Wu et al. [13], it was firstly demonstrated that the euthyroid patients with DN got lower FT3 levels compared with those without DN, which aroused interests to explore the correlation between serum FT3 and diabetic microvascular complications in euthyroid patients. Zou et al. [26] found that the prevalence of diabetic kidney disease exhibited a significant downward trend along the FT3 quartiles (41.1%, 30.6%, 23.8%, and 18.9%, p < 0.001). And compared with the first FT3 quartile group, the adjusted ORs [95% confidence interval (CI)] for diabetic kidney disease from the second to fourth FT3 quartile group were 0.655 (0.406–1.057), 0.493 (0.299–0.813), 0.406 (0.237–0.697). Likewise, Zou and his team further draw the similar conclusions on DR, that is, there was inverse correlation between FT3 within normal range and DR in T2DM patients [27]. However, it was still unknown that whether the correlation also existed between serum FT3 and DPN. Therefore, we carried out this cross-sectional study and found that the FT3 levels in DPN patients were remarkably lower than those in non-DPN patients, and the prevalence of DPN significantly decreased with the increasing FT3 quartile in euthyroid patients with type 2 diabetes. Moreover, after adjusting for confounding factors, the relation between FT3 quartiles and DPN remained significant in subjects from the first to third quartile relative to the fourth quartile, with ORs (95%CI) of 2.338 (1.407–3.884), 1.903 (1.134–3.194) and 1.598 (0.960–1.125), respectively. These findings further added the clinical evidence to the relations between FT3 levels and diabetic microangiopathy in euthyroid patients.

Studies regarding the association between FT3 levels and DPN are quite limited. Only one study by Zhu et al. [28] indicated that serum FT3 levels in normal nerve conduction group were statistically higher than those in abnormal nerve conduction group (4.55 ± 0.65 vs 4.37 ± 0.63, p < 0.05) for type 2 diabetic patients; however, they didn’t mention whether this association still existed in euthyroid patients with type 2 diabetes. Our results were consistent with the study by Zhu et al. [28] after we further extended the studied objects to euthyroid patients with type 2 diabetes. More interestingly, we found that a remarkable decrease in the prevalence of DPN was observed in middle-aged patients (age < 65) rather than older (age ≥ 65) patients, which suggested that our findings are more suitable for the middle-aged patients.

Nowadays, the underlying mechanisms on the association between FT3 and DPN remain elusive, the following enzymes and pathways may be involved in the development of DPN. Firstly, T3 directly and indirectly acted on the endothelial function in vitro by relaxing vascular smooth muscle [29]. The latest study showed that even small fibro neuropathy was related to damaged vascular endothelial function in type 2 diabetic patients [30]; therefore, low FT3 level and DPN may involve to the endothelial dysfunction. Secondly, T3 can facilitate progressive kidney impairment in db/db mice through significantly decreasing phosphatidylinositol 3-kinase activity and increasing the expression of transforming growth factor-β1 [31], and it also reported to accelerate the progression of DPN [32, 33]. Finally, in vivo and in vitro experimental models further showed that 3, 5-Diiodothyronine, a natural metabolite of T3, could ameliorate DN by regulating Sirtuin 1, which also played a vital role in prevention and reversal of DPN [34, 35].

Several studies have reported that there exists a powerful relation of FT3 to the presence and severity of CAD in euthyroid individuals [36, 37]. For example, Ertaş et al. [36] discovered that FT3 levels within the normal range were negatively related to the presence and severity of CAD for patients undergoing coronary angiography. Daswani et al. [37] also found that the genesis of CAD was in connection with lower serum FT3 levels; the concentrations of serum FT3 were also associated with the Gensini score which could make an independent prediction on the severity of CAD in euthyroid stable angina patients. However, studies regarding the associations between FT3 levels and macrovascular complications in euthyroid patients with type 2 diabetes are quite few. Recently, a study by Wang et al. [38] displayed that diabetic patients with low-normal FT3 level were more likely to suffer from macrovascular complications than those with mid-and-high normal FT3 level. Hu et al. [12] also revealed that there was a remarkable relation between diabetic macrovascular complications and normal FT3 (OR = 0.534, 95% CI 0.358–0.796). Partly consistent with the above studies, we found that patients in the lower FT3 quartiles were more prone to carotid atherosclerotic lesions than patients in the higher FT3 quartiles. But after controlling for confounders, we failed to show a significant association between FT3 quartiles and carotid atherosclerotic lesions. This discrepancy can be explained by the fact that the different definitions of macrovascular complications were used in above studies. That is, the macrovascular complications in the studies by Wang et al. [38] and Hu et al. [12] were defined as atherosclerosis from the aorta, coronary, basilar and carotid, while our current study mainly focused on carotid atherosclerotic lesions.

Additionally, we found that the incidence of carotid atherosclerotic lesions in type 2 diabetic patients with DPN was significantly higher than those without DPN, which suggested that DPN may be related to carotid atherosclerotic lesions in type 2 diabetes. This finding further provided strong evidence that diabetes-induced endothelial dysfunction was an important and initial factor in the development of diabetic vascular complications [39, 40].

The strength of our study as follows. To our best knowledge, it was the first study to investigate the relations of serum FT3 in normal range to DPN and carotid atherosclerotic lesion for euthyroid patients with type 2 diabetes. To early diagnosis and prevention of DPN, periodic screenings on thyroid hormones were recommended for euthyroid diabetic patients. Furthermore, our study fortified the powerful evidence for the hypothesis that the risk of diabetic microvascular complications may begin to increase when FT3 was at relatively low level. However, some limitations needed to be mentioned in our study. Firstly, further studies need to verify whether our results can be applied to other ethnic groups, other types of diabetes or community-based population as the participants in our report were Chinese Han inpatients with type 2 diabetes. Secondly, CIMT was used the IMT of the common carotid artery and other medications for the patients were not considered in our study expect medications affecting thyroid hormone levels. Thirdly, the indicators of thyroid function were measured once during the hospitalization, but they were obtained from all the patients after an overnight fast to avoid the differences of day-night variations in TSH from patients [41].

## Conclusions

In this study, we found that patients with low-normal FT3 levels were more likely to develop DPN than those with high-normal FT3 levels. And we also revealed that serum FT3 in normal range was inversely correlated with DPN but not with carotid atherosclerotic lesions in euthyroid patients with type 2 diabetes, independently of other risk factors. These findings indicated that low-normal FT3 may be a potential risk factor for DPN and provided the basis for the formulation of effective DPN prevention strategies. However, further prospective studies may need to verify the causal relations and mechanisms between FT3 and DPN in euthyroid patients with type 2 diabetes as it is a cross-sectional study.

## Supplementary Information

**Additional file 1: Table S1** Clinical characteristics of the subjects after adjusting for age and sex.

## Data Availability

The datasets used and/or analysed during the current study are available from the corresponding author on reasonable request.

## Abbreviations

CAD: Coronary artery disease
SCH: Subclinical hypothyroidism
DN: Diabetic nephropathy
DR: Diabetic retinopathy
DPN: Diabetic peripheral neuropathy (DPN)
TSH: Thyroid stimulating hormone
FT4: Free thyroxine
FT3: Free triiodothyronine
BMI: Body mass index
WHR: Waist hip ratio
SBP: Systolic blood pressure
DBP: Diastolic blood pressure
HbA1c: Glycated hemoglobin A1c
FPG: Fasting plasma glucose
2 h PPG: 2 H postprandial plasma glucose
FCP: Fasting C-peptide
2 h PCP: 2 H postprandial C-peptide
HOMA-IR: Homeostasis model assessment for insulin resistance
TTG: Total triglycerides
TC: Total cholesterol
HDL-C: High-density lipoprotein cholesterol
LDL-C: Low-density lipoprotein cholesterol
ALT: Alanine aminotransferase
Cr: Creatinine
SUA: Serum uric acid
CRP: C-reactive protein
UACR: Urine albumin-to-creatinine ratio
eGFR: Glomerular filtration rate
NCV: Nerve conduction velocity
CIMT: Carotid intima-media thickness
ORs: Odds ratios
DD: Duration of diabetes
95% CI: 95% Confidence interval
