# Supplementary material for: Serum free triiodothyronine is inversely associated with diabetic peripheral neuropathy but not with carotid atherosclerotic lesions in euthyroid patients with type 2 diabetes
Source: Diabetol Metab Syndr. 2021 Dec 4;13:142. doi: 10.1186/s13098-021-00760-2 (PMC8645151; doi:10.1186/s13098-021-00760-2)
Supplement: Supplementary file 1 — Additional file 1: Table S1 Clinical characteristics of the subjects after adjusting for age and sex. [file 13098_2021_760_MOESM1_ESM.docx]

**Additional file 1**

**Table S1 Clinical characteristics of the subjects after adjusting for age and sex**

| **Variables** | **Q1(n=612)** | **Q2(n=616)** | **Q3(n=622)** | **Q4(n=627)** | **p value** |
| --- | --- | --- | --- | --- | --- |
| FT3 (pmol/L) | 3.10-4.10 | 4.10-4.40 | 4.40-4.72 | 4.72-6.80 | - |
| Age (years) | 64±11 | 61±11 | 58±12 | 55±11 | <0.001 |
| Men (n, %) | 261(42.6%) | 298(48.4%) | 372(59.8%) | 459(73.2%) | <0.001 |
| *DD (months) | 120(48-180) | 96(48-156) | 90(36-144) | 72(24-120) | 0.376 |
| Smoking (n, %) | 114(18.6%) | 133(21.6%) | 160(25.7%) | 241(38.4%) | 0.339 |
| Alcohol (n, %) | 48(7.8%) | 71(11.5%) | 85(13.7%) | 114(18.2%) | 0.346 |
| Hypertension (n, %) | 314(51.3%) | 330(53.6%) | 300(48.2%) | 270(43.1%) | 0.171 |
| BMI (kg/m^2^) | 24.63±3.68 | 25.28±3.38 | 25.08±3.45 | 25.41±3.38 | 0.030 |
| WHR | 0.93±0.35 | 0.92±0.07 | 0.91±0.06 | 0.92±0.06 | 0.238 |
| SBP (mmHg) | 133±18 | 132±17 | 132±18 | 131±16 | 0.074 |
| DBP (mmHg) | 79±10 | 79±10 | 80±9 | 82±9 | 0.007 |
| *FPG (mmol/l) | 7.55(6.04-9.82) | 7.82(6.31-10.08) | 7.90(6.26-9.69) | 7.81(6.40-9.63) | 0.059 |
| 2 h PPG (mmol/l) | 14.13±4.96 | 14.07±4.66 | 13.89±4.66 | 13.67±4.72 | 0.017 |
| *HbA1c (%) | 9.3(7.5-11.5) | 8.7(7.3-10.5) | 8.5(7.3-10.2) | 8.4(7-9.9) | <0.001 |
| *FCP (ng/mL) | 1.57(0.89-2.40) | 1.73(1.10-2.54) | 1.82(1.20-2.53) | 1.89(1.30-2.53) | 0.014 |
| 2 h PCP (ng/mL) | 3.75±2.87 | 4.15±2.74 | 4.46±3.01 | 4.75±2.71 | <0.001 |
| *HOMA-IR | 4.94(2.98-8.40) | 4.88(2.96-7.85) | 4.59(2.91-7.75) | 4.40(2.72-7.40) | 0.854 |
| *TTG (mmol/L) | 1.38(0.92-2.08) | 1.50(1.01-2.18) | 1.54(1.04-2.41) | 1.51(1.09-2.10) | 0.009 |
| TC (mmol/L) | 4.88±1.35 | 4.84±1.33 | 4.79±1.04 | 4.67±0.99 | 0.009 |
| HDL-C (mmol/L) | 1.17±0.40 | 1.14±0.31 | 1.10±0.31 | 1.10±0.28 | 0.409 |
| LDL-C (mmol/L) | 3.16±1.03 | 3.18±0.98 | 3.24±0.93 | 3.17±0.92 | 0.173 |
| *ALT (U/l) | 17(12-26) | 19(13-29) | 20(14-30) | 20(14-30) | 0.093 |
| *Scr (μmol/l) | 63(52-80) | 65(55-77) | 65(55-78) | 67(56-78) | <0.001 |
| *SUA (μmol/l) | 310(251-369) | 300(248-364) | 310(265-377) | 319(267-377) | 0.911 |
| *UACR (mg/g) | 13.05(6.56-40.28) | 11.73(6.77-32.96) | 11.25(6.51-27.99) | 10.14(5.99-24.68) | 0.002 |
| *eGFR(ml/min/1.73m^2^) | 95.35(76.97-118.23) | 101.37(83.68-117.81) | 102.78(86.18-120.92) | 108.25(92.07-126.77) | 0.226 |
| *CRP(mg/L) | 1.46(0.53-3.87) | 1.09(0.54-2.61) | 0.96(0.47-2.12) | 0.93(0.45-1.87) | <0.001 |
| FT4 (pmol/L) | 15.96±2.19 | 16.01±2.20 | 16.34±2.19 | 16.74±2.21 | <0.001 |
| TSH (mIU/L) | 1.74±0.89 | 1.74±0.81 | 1.69±0.84 | 1.67±0.85 | 0.655 |

Values are expressed as the mean ± standard deviation, median with interquartile range, or percentages.

*Non-normal distribution of continuous variables.

Abbreviations: FT3, free triiodothyronine; DD, duration of diabetes; BMI, Body mass index; WHR, Waist hip ratio; SBP, Systolic blood pressure; DBP, Diastolic blood pressure; FPG, Fasting plasma glucose; 2 h PPG, 2 h Postprandial plasma glucose; HbA1c, glycosylated haemoglobin A1c; FCP, fasting C-peptide; 2 h PCP, 2-h postprandial C-peptide; HOMA-IR, Homeostatic model assessment of insulin resistance; TTG, Total Triglyceride; TC, Total cholesterol; HDL-C, High-density lipoprotein cholesterol; LDL-C, Low-density lipoprotein cholesterol; ALT, alanine aminotransferase; Scr, serum creatinine; SUA, serum uric acid; UACR, urine albumin-to-creatinine ratio; eGFR, estimated glomerular filtration rate; CRP, C-reactive protein; FT4, free thyroxine; TSH, thyroid stimulating hormone.

**Supplemental Table 2 The Spearman’s correlation coefficients among independent variables associated with DPN**

**
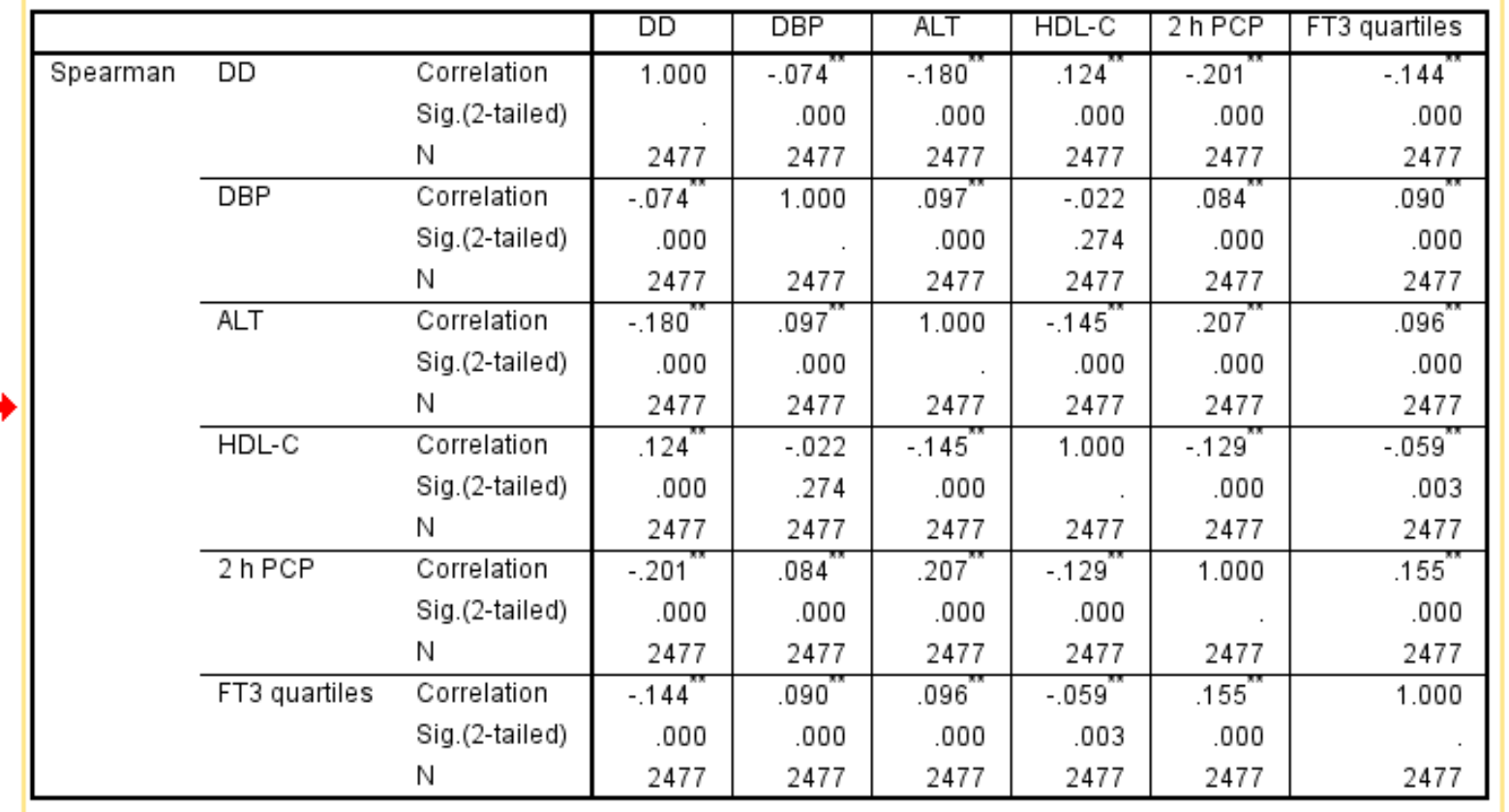
**

**Supplemental Table 3 Collinearity diagnosis among independent variables associated with DPN**

**
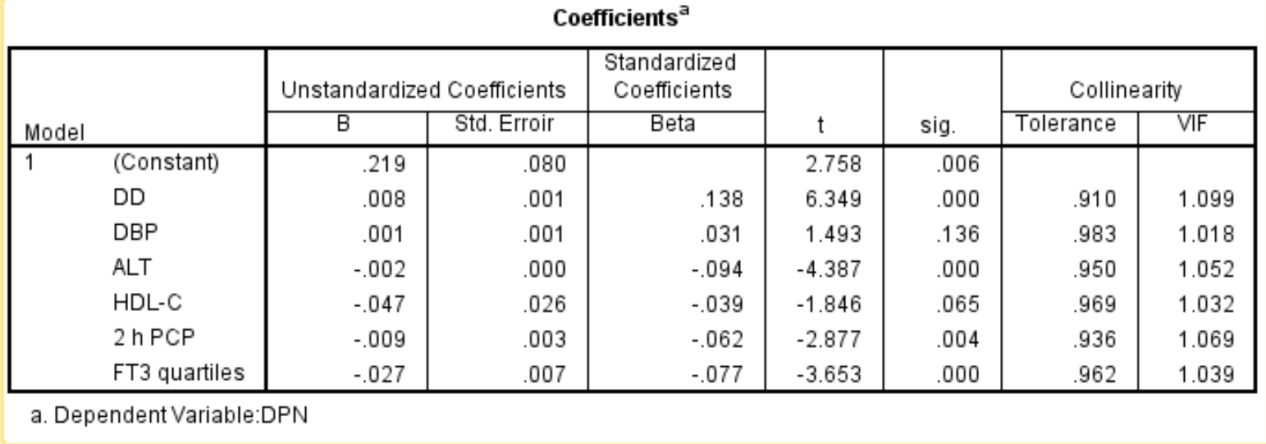
**
